# Preventable deaths following emergency medical dispatch – an audit study

**DOI:** 10.1186/s13049-014-0074-y

**Published:** 2014-12-19

**Authors:** Mikkel S Andersen, Søren Paaske Johnsen, Andreas Ernst Hansen, Eivinn Skjaerseth, Christian Muff Hansen, Jan Nørtved Sørensen, Søren Bruun Jepsen, Jesper Bjerring Hansen, Erika Frischknecht Christensen

**Affiliations:** Research Department, Prehospital Emergency Medical Services, Aarhus, Central Denmark Region, Olof Palmes Allé 34, 8200 Aarhus N, Denmark; Department of Clinical Epidemiology, Aarhus University Hospital, Aarhus, Denmark; Department of Anesthesiology, Aarhus University Hospital, Aarhus, Denmark; Emergency Medical Communication Center, Oslo University Hospital, Oslo, Norway; Helicopter Emergency Medical Services, St. Olavs Hospital, Trondheim, Norway; Department of Anaesthesia, Center of Head and Orthopedics, Rigshospitalet, University of Copenhagen, Copenhagen, Denmark; Emergency Medical Communication Center, Capital Region of Denmark, Denmark; Mobile Emergency Care Unit, Department Anaesthesiology Intensive Care Medicine, Odense University Hospital, Odense, Denmark

**Keywords:** Emergency medical dispatch, Audit, Preventable deaths

## Abstract

**Background:**

Call taker triage of calls to the 112 emergency number, can be error prone because rapid decisions must be made based on limited information. Here we investigated the preventability and common characteristics of same-day deaths among patients who called 112 and were not assigned an ambulance with lights and sirens by the Emergency Medical Communication Centre (EMCC).

**Methods:**

An audit was performed by an external panel of experienced prehospital consultant anaesthesiologists. The panel focused exclusively on the role of the EMCC, assessing whether same-day deaths among 112 callers could have been prevented if the EMCC had assessed the situations as highly urgent. The panels’ assessments were based on review of patient charts and voice-log recordings of 112 calls. All patient related material was reviewed by the audit panel and all cases where then scored as preventable, potentially preventable or non-preventable during a two day meeting. The study setting was three of five regions in Denmark with a combined population of 4,182,613 inhabitants, which equals 75% of the Danish population. The study period was 18 months, from mid-2011 to the end of 2012.

**Results:**

Linkage of prospectively collected EMCC data with population-based registries resulted in the identification of 94,488 non-high-acuity 112 callers. Among these callers, 152 (0.16% of all) died on the same day as the corresponding 112 call, and were included in this study. The mean age of included patients was 74.4 years (range, 31–100 years) and 45.4% were female. The audit panel found no definitively preventable deaths; however, 18 (11.8%) of the analysed same-day deaths (0.02% of all non-high-acuity callers) were found to be potentially preventable. In 13 of these 18 cases, the dispatch protocol was either not used or not used correctly.

**Conclusion:**

Same-day death rarely occurred among 112 callers whose situations were assessed as not highly urgent. No same-day deaths were found to be definitively preventable by a different EMCC call assessment, but a minority of same-day deaths could potentially have been prevented with more accurate triage. Better adherence with dispatch protocol could improve the safety of the dispatch process.

## Introduction

Emergency Medical Dispatch is in essence assessment of emergency calls, decision on the level of emergency, giving medical advice and allocation of ambulances and specialised prehospital units and it constitutes an essential part of the chain of survival.

In the Emergency Medical Communication Centres (EMCC) performing Emergency Medical Dispatch, call taker triage of patients is susceptible to errors since decisions must be made rapidly based on limited information. Such errors can impact patient outcome, resulting in increased morbidity or death. Previous studies have investigated the preventability of deaths among acutely ill and injured patients admitted to emergency departments and among trauma patients [1-4]. Only one previous audit study examined, as a secondary endpoint, the preventability of death following Emergency Medical Dispatch [5].

The last three years have seen considerable changes to the organization of Emergency Medical Dispatch in Denmark. The system was formerly mainly police operated—but with the opening of EMCCs staffed with nurses, paramedics, and doctors to assess all 112 calls, it has become an integrated part of public health care. Ten years ago, a survey of prehospital care in the Nordic countries highlighted the former police-operated system as a weak link in Danish prehospital care [6]. A statement that was stressed by studies showing moderate to low accuracy in detection of acute coronary syndrome patients and patients with loss of consciousness [7,8]. The Danish EMCCs assess all incoming calls using a criteria-based dispatch protocol (the Danish Index for Emergency Care), which divides patients into five levels of emergency (A–E) based on their main symptoms [9-11]. Criteria-based dispatch constitutes a decision support and registration system operated by healthcare professionals as opposed to the strict protocol used in Medical Priority dispatching operated by non-healthcare professionals. The recent reorganization was intended to improve the initial assessment and triage of callers to the 112 emergency telephone number; however, it has not yet been evaluated whether this goal has been accomplished.

The first study of the new EMD system in Denmark revealed a group of 112 callers who were not assessed as highly urgent but who died on the day of their 112 call [10]. These patients potentially represent serious undertriage and, therefore, warrant further more detailed investigation. The primary objective of the present study was to determine the proportions of preventable and potentially preventable same-day deaths among the 112 callers who were not assessed as highly urgent. Secondly, we wanted to identify common characteristics among preventable deaths, in order to detect areas for improvement of the EMD process.

## Methods

### Setting

In Denmark, criteria-based EMD is conducted in five regional EMCCs. The common number for fire, police, and health-related emergencies is 112, which is answered by the police (or by the fire brigade in part of the capital area). Following establishment of the caller’s geographical position, all calls concerning illness and injury are redirected to an EMCC. According to the Danish Index for Emergency Care (Danish Index), the EMCC staff categorizes calls into one of 37 chief complaint groups that are each subdivided into five levels of emergency: A: life-threatening or potentially life-threatening condition requiring immediate response (“blue lights and sirens”); B: urgent but not life-threatening condition; C: non-urgent condition that requires an ambulance; D: non-urgent condition requiring supine patient transport; and E: condition requiring other service or advice/instruction, including taxi transportation (no ambulances are dispatched for emergency level E calls). Each level of emergency contains a number of more specific symptoms, each with a specific index code. Ambulance response times differ somewhat between the regions, but for the majority of emergency level A the target value is max 10 min, level B max 15–20 min, C and D max 90 min. The emergency level A ambulance turnouts are in many cases accompanied by a physician staffed medical emergency unit (MECU).

In addition to the 112 system there is a general practitioner ‘on call’ system in Denmark available 24 hours a day. One region (Capital) established in February 2012 a telephone line (1813) for less urgent medical calls. Ambulance dispatches arising from any of these systems were not included in this study.

### Population and study design

This study was conducted in the three largest regions of the country (Central, Southern, and Capital), which have a combined population of 4,182,613 inhabitants (1st of January, 2012), equalling 75% of the total Danish population (n = 5,580,516) [12]. The study population comprised all 112 callers who were registered by the EMCCs as emergency level B–E, and who died on the same date as the corresponding emergency call. The study period was from July 1st 2011 to the end of 2012 (18 months).

The study was designed as a medical audit performed by an external expert panel and carried out as a retrospective review of all patient-related material as described by Lembcke et al., Mainz et al., and Nakano et al. [13-15]. The expert panel consisted of three consultant anaesthesiologists with extensive prehospital experience and with no affiliation to the evaluated EMCCs.

### Data sources

**Figure 1:**
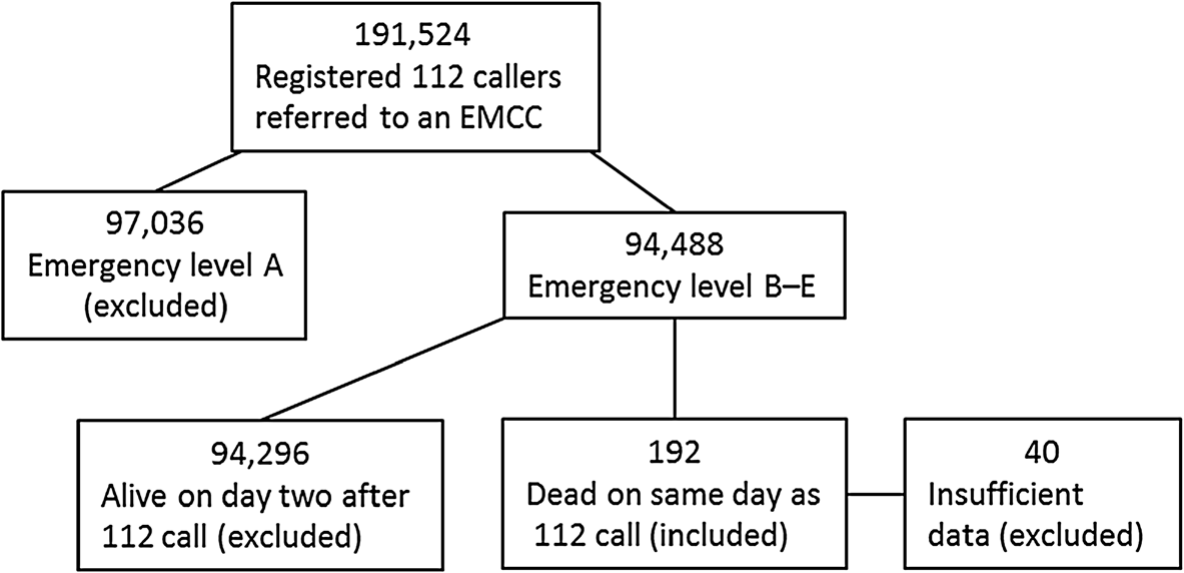
**Flowchart of patients included in and excluded from the study.**

Patients were identified through the EMCC dispatch software. All contacts to the EMCC from the 112 system were identified, and the patient’s civil registration number and Danish Index code (including level of emergency) were documented. The unique 10-digit civil registration number is assigned to all Danish residents at birth or immigration; it allows unambiguous linkage between all Danish medical and administrative registers [16]. From the Civil Registration System, we obtained information on age, gender, and change in vital status (dead or alive). Patients who received a Danish Index code consistent with emergency level B or lower *and* who died on the same date as their 112 call, were included in the present investigation (Figure 1). For each included patient, we recorded the patient’s prehospital time interval, which included the EMD response interval (from call pick up at the EMCC until dispatch of the first ambulance) and the EMS response interval (from ambulance dispatch to arrival on scene). We utilized the National Registry of Patients (NRP) to obtain data on hospital admissions. The NRP hold records of 99.4% of all discharges from Danish hospitals since 1977, and on all in- and out-patient hospital visits since 1995 [17]. For each patient, we retrieved the prehospital medical records from the ambulance services, the record from the involved hospital, and the post-mortem report when available. From the involved EMCCs, we also obtained voice log recordings of the telephone conversations between the 112 callers and the EMCC. If patient charts were missing or not filled in, patients were excluded from the study.

### The audit process

All material was retrieved and reviewed by a member of the study group (Andersen MS). After initial review patients with inadequate and missing information on circumstance surrounding their death were excluded (Figure 1). Summaries of all included deaths were produced by Andersen MS. Table 1 presents the content of the patient summary. Material relating to the deaths—including all charts, voice logs, post-mortems, and summaries—was uploaded to a secure server accessible only to the expert panel. The external reviewers (Hansen AE, Skjaerseth E, Hansen CM) were asked to evaluate the material before a two-day meeting, during which all death-related material was jointly reviewed with Andersen MS as the facilitator.

**Table 1:** **Information included in patient summary used by audit panel**

1.	Date and time of 112 call referred to EMCC:
2.	Danish Index criteria (index code):
3.	Additional information, if any, in dispatch software visible in ambulance:
4.	EMD response interval (min:sec):
5.	EMS response interval (min:sec):
6.	Vital signs:
7.	Summary of pre- and in-hospital charts and tests results:
8.	Short summary of prior medical history (if any):
9.	Age:
10.	Time of death:

Min, minutes; sec, seconds.

The reviewers were asked to determine whether each patient’s death was preventable, potentially preventable, or non-preventable. In the judgement of preventability, the reviewers were asked to state what the EMCC should or could have done differently. The reviewers were instructed to exclusively focus on factors related to the EMCC call-taker. Preventable death in this study was defined as a death that could have been prevented if the EMCC had assessed the call differently, such that an ambulance with blue lights and sirens was dispatched to the patient, as well as a supplemental prehospital doctor in the most severe cases. The quality of care provided by ambulance staff, and prehospital or in-hospital doctors was not subjected to review in this study. Preventability was determined according to the experts’ professional judgment of each included death, based on thorough review of all available patient-related material before and during the two-day audit meeting, as well as on the reviewers’ considerable experience with prehospital emergency care. A death was deemed preventable if there was a high certainty that a different assessment by the EMCC could have prevented the death. Potentially preventable deaths were cases in which a different assessment by the EMCC could have potentially prevented the death. These preventability categories were inspired by audit studies by Kuisma et al., Lu et al., and Nafsi et al. [1,2,5]. Any dissent between the three experts was resolved by discussion and, if any disagreement remained, it was settled with majority decision.

### Statistics

Descriptive statistics of the included patients were presented. No formal statistical comparisons or tests were made.

### Ethics

This study was approved by the Danish Data Protection Agency (reference number 2011-41-6326 and 2013-41-1598). Permission to inspect patient charts was granted by the Danish Health and Medicines Authority (ref. nr.3-3013-257/1/). Permission from the Ethics Committee is not required for studies using routinely accumulated data according to Danish law.

## Results

During the study period, a total of 314,134 calls to the 112 number were redirected to the three EMCCs included in the study. Of these callers, 191,524 were registered in the EMCC dispatch software with a valid civil registration number and Danish Index code. A total of 94,488 were assessed as emergency level B–E. Of these callers, 192 (0.2%) died on the same date as calling the 112 number and were hence eligible for review. Forty of these deaths were excluded due to insufficient information. One half of the exclusions were due to a failed filing system of prehospital records in one region during the first part of the study period. The other half were due to very sparse information noted on pre- and in-hospital charts combined with missing voice-log recordings, which together left too little information available to assess preventability. A total of 152 deaths were included in the study, comprising 0.16% of all B–E callers. The mean age of the callers at the time of death was 74.4 (range, 31–100) and 45.4% were female. Table 2 displays the distribution of included patients according to the Danish Index level of emergency and chief complaint groups. The majority was assessed as urgency level B, and the remainder as lower emergencies of which one patient was emergency level E. The most frequently entered chief complaint was “unclear problem” (66 of 152) followed by “difficulty in breathing” (27 of 152).

**Table 2:** **Distribution of Danish Index chief complaint groups among the included patients**

**Chief complaint group**	
**Emergency level B**	**n**
Unclear problem	64
Difficulty in breathing	27
Stomach or back pain	15
Minor wound, fracture, injury	10
Seizure	6
Accident (not traffic related)	6
Impaired consciousness, paralysis	6
Chest pain, heart disease	3
Poisoning, medications, alcohol, drugs	3
Urinary system	3
Bleeding, non-traumatic	2
Diabetes	2
Emergency level C	
Difficulty in breathing	3
Unclear problem	1
Emergency level E	
Unclear problem	1
Total	152

None of the 152 deaths included in the study were considered definitively preventable by any of the reviewers. Eighteen of the included deaths (11.8% of the included deaths and 0.02% of total B–E callers) were considered potentially preventable *if* the EMCC had assessed the 112 call as more urgent and this had led to an ambulance dispatch with a shorter response time and possible rendezvous with a physician-staffed mobile emergency care unit (MECU). The reviewers rated 134 deaths (88.2%) as non-preventable. One example of a non-preventable death was that of a 91-year-old female assessed by the EMCC as emergency level B due to stomach pain. At the hospital, the patient was awake and orientated with normal vital signs. The patient was diagnosed with a ruptured abdominal aortic aneurism and declined further treatment. The patient died at the hospital 7 hours after the 112 call. Another non-preventable death was that of a 56-year-old male who was assessed by the EMCC to be emergency level B after a minor seizure. The patient was admitted to the hospital where he recovered to his normal state with normal vital signs; however, eight hours later, the patient developed hematemesis and died.

The median EMD response interval was 3 min 26 sec among the potentially preventable deaths and 3 min 20 sec among the non-preventable deaths. The median EMS response interval was 12 min 23 sec among the potentially preventable deaths and 9 min 25 sec among the non-preventable deaths. Table 3 presents the characteristics of the patients who experienced potentially preventable or non-preventable deaths and of the survivors.

**Table 3:** **Potentially preventable vs. non-preventable deaths**

	**Potentially preventable**	**Non-preventable**	**Emergency level B–E survivors**
**Characteristics**	**(n = 18)**	**(n = 134)**	**(n = 94,336)**
Female, n (%)	6 (33.3)	63 (47.0)	47,074 (49.8)
Age, mean (range)	66 (34–88)	76 (31–100)	53 (0–101)
EMD time in min:sec, median (IQR)	3:26 (3:00–5:26)	3:20 (2:10–5:11)	3:25 (2:19–5:36)
EMS time in min:sec, median (IQR)	12:23 (7:20–15:28)	9:25 (6:32–13:31)	9:51 (6:34–15:03)

Min, minutes; sec, seconds.

The potentially preventable deaths fell in two groups. In one group (n = 5), the EMCC call-takers, in principle, reacted adequately to the inquiry based on the content of the telephone interview; however, it later turned out that a different response could have benefitted the patient. One example of such a death occurred in a 62-year-old female with difficulty breathing. Her husband was the caller, and it was possible to hear the patient talk and yell in the background. The EMCC nurse chose a priority B “difficulty breathing, gradually deteriorating” criterion. At ambulance arrival, the patient was cyanotic and in severe respiratory distress. The patient went into cardiac arrest and a MECU was summoned and arrived after 10 minutes. Fifty-two minutes after the onset of cardiac arrest, the patient was declared dead in the emergency department. The review panel concluded that the EMCC nurse reacted adequately according to the Danish Index and the content of the telephone interview, but that the immediate dispatch of an emergency level A ambulance and a MECU could potentially have prevented the fatal outcome for the patient.

In the second group of potentially preventable deaths (n = 13; 0.01% of total B–E callers), the expert panel determined that either the Danish Index was not used or it was used incorrectly by the call-taker. An example of a potentially preventable death in this second group was that of a 77-year-old female who was found on the floor by her son. On the voice log, the patients’ son is heard to inform the EMCC that his mother might have a broken arm and to mention twice that she had severe breathing difficulties. An ambulance was dispatched as emergency level B under the criterion of a possible fracture. At ambulance arrival, the patient was in cardiac arrest. At that time, the ambulance staff summoned a MECU staffed with an experienced anaesthesiologist with prehospital emergency medical training. The MECU arrived 24 minutes after the 112 call, and the patient was declared dead 8 minutes later. The expert panel determined that the patient should have been assigned a “difficulty breathing” criterion and assessed as emergency level A. They concluded that a joint response with ambulance and MECU was justified based on the content of the 112 call, and that such a response could have potentially prevented the fatal outcome.

Among the potentially preventable deaths, most EMD response intervals were between 1 and 4 minutes. In two cases, the EMD response interval was above 10 minutes. The EMS response intervals were between 6 and 13 minutes in most cases. In four cases, the EMS response intervals were between 17 and 38 minutes. Table 4 presents information on all potentially preventable deaths.

**Table 4:** **Summary of potentially preventable deaths**

**Patient**	**Danish Index chief complaint**	**Age (sex)**	**EMD response interval**	**EMS response interval**
1	Unclear problem	63 (M)	03:44	13:47
2	Unclear problem	66 (F)	04:17	12:43
3	Unclear problem	69 (M)	12:01	06:41
4	Unclear problem	83 (M)	02:08	06:45
5	Unclear problem	74 (M)	03:26	07:33
6	Unclear problem	61 (M)	07:37	08:27
7	Unclear problem	73 (M)	05:08	12:25
8	Difficulty in breathing	56 (F)	01:41	12:32
9	Difficulty in breathing	62 (F)	03:36	06:34
10	Difficulty in breathing	34 (F)	04:31	17:42
11	Difficulty in breathing	68 (F)	11:19	15:47
12	Chest pain, heart disease	74 (M)	.	13:00
13	Chest pain, heart disease	88 (M)	01:51	38:31
14	Poisoning, medications, alcohol, drugs	44 (M)	15:33	24,45
15	Poisoning, medications, alcohol, drugs	70 (M)	03:00	04:10
16	Seizure	52 (M)	01:39	17:58
17	Accident (not traffic related)	77 (F)	03:24	11:09
18	Urinary system	76 (M)	05:43	12:51

## Discussion

Same-day deaths occurring among 112 callers who were not assessed by the EMCC to have a life-threatening condition could represent very serious undertriage. The present independent expert review of telephone call recordings and patient charts found that none of these same-day deaths were definitively preventable with high certainty—i.e. in no case was there a high probability that the death could have been avoided if the EMCC had made a different assessment. Our review identified a number of potentially preventable deaths that could possibly have been averted if the EMCC had made a different call assessment; however, these constituted a very small proportion of all non-high-acuity patients (one potentially preventable death for every 5,249 non-high-acuity 112 caller). The majority of cases in which death was deemed potentially preventable involved incorrect use or no use of dispatch protocol. Most of the potentially preventable deaths occurred with an EMS response interval of around 13 minutes or less. These were not extremely long intervals, but they would likely have been markedly shorter if the calls had been assessed as emergency level A and “blue lights and sirens” had been used. Four of the potentially preventable deaths showed EMS response intervals of 17 minutes and up to 38 minutes, which constitute time-spans that may have substantially influenced patient prognosis.

One earlier study investigated preventability of death occurring in close relation to a 112 call [5]. Among deaths occurring in lower-priority groups in Finland, Kuisma et al. reported that 1.3% were preventable, 32.9% were potentially avoidable, and 65.8% were non-preventable. These proportions of preventable and potentially preventable deaths are markedly higher compared to our present findings; however, it is unclear whether the review process and definitions of preventability were the same as in our study. The previously published chart review was a secondary aim of a Finnish study, and thus the audit process was not described in detail. A number of other audit studies have investigated early mortality related to emergency departments and trauma centres. Lu et al. performed a chart review of deaths occurring within 24 hours after admission to an in-house ward from the emergency department (ED) [2]. They found that 25.8% of early deaths were preventable. In an audit with external patient chart review, Nafsi et al. evaluated deaths that occurred within 7 days of admission to an ED, and found that 3.15% were definitely preventable and 9.46% were either possibly or probably preventable [1]. In a Dutch trauma centre audit, Saltzherr et al. reported that 2% of deaths were preventable and 27% were potentially preventable [3]. Compared to these previous studies, our present results were fairly good, with zero definitively preventable deaths and 11.8% potentially preventable deaths. This comparison must take into account the longer duration of patient contact in an ED admission compared to the short prehospital time interval. A higher proportion of deaths are likely to occur due to suboptimal treatment during the hours or days of a hospital admission than as a consequence of actions during the shorter time from a 112 call until the arrival of an ambulance and/or doctor to the patient.

For investigating whether deaths or other adverse events are avoidable, a well-planned chart review by an expert panel is a reliable method that also provides opportunity for identifying possible areas of future improvement [1,2,13,14,18]. One limitation of the present study was the incomplete registration of civil registration numbers and Danish Index codes into the EMCC software. We examined the rate of missing data in smaller clusters (e.g. comparing between the three EMCCs and between shorter time periods), which revealed no indications of selection bias. Another limitation of the study was the exclusion of some patients whose pre- and in-hospital charts were missing or insufficient, which could introduce selection bias. Since the data registration in this newly implemented EMD system is incomplete, it is not possible to guaranty that all deaths are accounted for in this study. However, the authors had no reason to believe that the group of excluded patients contained a higher proportion of preventable deaths than the included patients since all data at the EMCCs were prospectively registered. Therefore the EMCCs had no knowledge about the later death of the patients at the time the data should be registered.

The non-emergency general practitioner services and ambulance dispatches arising from this system were not included in the study. This was the case mainly because these services do not use the Danish Index for Emergency Care, which was the focus of our study. Furthermore these services are organized in different ways around the country making the availability and quality of data heterogenic.

The inclusion of only deaths occurring on the same date may constitute a limitation, since it does not include e.g. 112 calls put at 11:00 pm concerning patients who subsequently dies at e.g. 01:00 am on the following date. The inclusion of only same date deaths was mainly due to the way deaths are registered in the civil registration system, where only data on the date and not time of day is registered.

In daily clinical practice at the EMCC, it is a general impression that 112 calls that end with a suboptimal outcome for the patient or a complaint from the caller are often the result of the dispatcher failing to comply with the dispatch protocol. This impression was confirmed by the present study, as 13 of the 18 potentially preventable deaths were associated with non-compliance with the dispatch protocol. In a study of the Norwegian criteria-based dispatch protocol, Ellensen et al. reported large variations between the EMCCs regarding adherence to the dispatch protocol [19]. On average, the Norwegian dispatch protocol was followed by call-takers in 75% of calls. In a Norwegian study of EMCC handling of calls concerning intoxication, Lorem et al. reported that 89% of dispatchers used the CBD protocol, but that 33% of the calls included in the study showed deviations from the protocol [20].

Our present findings that none of the same-day deaths among non-high-acuity 112 callers were considered preventable and that few were potentially preventable, are encouraging results regarding the new EMD system in Denmark—especially when considering the young age of the system, and the almost 200,000 calls that this study was based on. A limited number of patients among the potentially preventable deaths may have suffered serious consequences of the EMCC triage. There is room for improvement in terms of systematic protocol adherence.

## Conclusion

The incidence of fatal adverse outcomes when an emergency medical dispatch protocol is used, was very low. No preventable same-day deaths were identified among non-high-acuity 112 callers. A small proportion of same-day deaths among all non-high-acuity 112 callers were assessed as potentially preventable by audit panel. Better alignment with dispatch protocol may further improve the safety of the dispatching process.
